# Assessment of the Effectiveness of Aloe vera Versus Amlexanox in the Treatment of Recurrent Aphthous Ulcers: A Three-Arm Placebo-Controlled Randomized Clinical Trial

**DOI:** 10.7759/cureus.30693

**Published:** 2022-10-26

**Authors:** Nasem Jamal Yousef, Abeer Ahmad Aljoujou, Ammar Mahmoud Mashlah, Mohammad Y Hajeer

**Affiliations:** 1 Department of Oral Medicine, University of Damascus Faculty of Dentistry, Damascus, SYR; 2 Department of Orthodontics, University of Damascus Faculty of Dentistry, Damascus, SYR

**Keywords:** ulcer healing, pain assessment, effectiveness, oral mucosa, treatment, ulcer size, amlexanox, aloe vera, recurrent aphthous ulcers

## Abstract

Background

Recurrent aphthous ulcers are one of the most common lesions of the oral mucosa. Most currently available treatment methods aim to relieve symptoms, speed up healing and prevent ulcer recurrence. The current study aimed to compare the effectiveness of *Aloe vera* gel with that of amlexanox 5% oral paste in the treatment of recurrent small-type aphthous ulcers.

Materials and Methods

The study was conducted on 60 patients (27 males and 33 females) attending the Department of Oral Medicine at the Faculty of Dentistry at Damascus University. The sample age ranged between 15 to 25 years, with an average age of 20.3 ± 2.4 years. Patients were diagnosed with recurrent aphthous ulcers of the small type. The sample was divided into three groups with equal numbers of patients (n=20 for each group) according to the provided drug: Aloe vera, amlexanox, and the placebo groups. Patients' ulcer size was measured on day 0 of treatment, and the ulcer size reduction was assessed on day 3 and day 5. The pain was also recorded at the first visit, and then pain reduction was assessed during follow-up visits.

Results

The mean ulcer size on the fifth day of treatment was 1.85 mm^2^, 4.05 mm^2^, and 6.20 mm^2^ in the Aloe vera, the amlexanox, and the placebo groups, respectively. The differences between groups were significant (p=0.003). The mean pain on the fifth day was 0.80 cm, 1.60 cm, and 3.20 cm in the Aloe vera, the amlexanox, and the placebo groups, respectively. The differences between groups were significant (p=0.026).

Conclusions

Within the limits of the current trial, both treatment groups proved effective in accelerating ulcer healing with the superiority of Aloe vera compared to amlexanox, as it achieved a greater reduction in ulcer size and pain when assessed on the fifth day of treatment.

## Introduction

Recurrent aphthous ulcers are a common cause of oral ulcers. The etiology of these lesions that occur only in the oral mucosa is unknown [1]. It is associated with stress factors, physical or chemical trauma, food allergy, and genetic predisposition [2]. Aphthous ulcers do not have clear causes, and sometimes they are difficult to manage and cause inconvenience to patients [3]. The recurrence of these ulcers can be due to genetic, psychological, physical, infectious, and hormonal factors such as pregnancy or post-menopausal factors, trauma, stress, food allergies, nutritional deficiency, iron and vitamin B12 deficiency, folic acid, and hematological abnormalities [2].

Aphthous ulcers are divided into three clinical types: minor and major aphthous ulcers and herpetiform ulcers. Minor aphthous ulcers, the most common, are small in size (less than 5 mm in diameter) and have a form of a single ulcer or several ulcers [4,5]. Major aphthous ulcers are less common, usually with a diameter of 5 mm or larger, and have single or multiple forms. These aphthous ulcers can be painful, especially when eating or drinking, and lasts between two weeks and several months. Dysphagia is associated with the site of the lesion, being most common in the inner mucosa of the lip, tongue, and soft palate [5]. The third type is herpetiform, which is rare. It has a size of 0.1-0.2 cm and is presented in large numbers. It can appear as a large, irregular lesion with a clinical appearance of 7-14 days. This ulcer occurs when several small ulcers fuse to form large, irregularly shaped ulcers [5]. Treatment methods for these aphthous ulcers include using nutritional supplements, local anesthetics, antiseptics, and steroids. In severe cases, systemic immune modulators and corticosteroids are pharmacological agents with varying efficacy and side effects. The diagnosis of recurrent aphthous ulcers is mainly based on the medical history and clinical examination [6,7]. The treatment of aphthous ulcers varies among age and population groups. Patients with mild recurrent aphthous ulcers usually do not require any treatment for the lesion [8]. But when the treatment is recommended, there are many ways to treat recurrent aphthous ulcers, such as systemic treatments, topical agents, physical treatments, and laser treatments [9].

Topical agents are the first choice for the management of recurrent aphthous ulcers. They are cost-effective, safe, and readily available [1,10]. Amlexanox (C16H14N2O4) is one of the most widely studied topical agents used to treat recurrent aphthous ulcers [11]. It has an anti-inflammatory and anti-allergic effect as it inhibits the formation and release of histamine and leukotriene from mast cells, neutrophils, and mononuclear cells [9]. *Aloe vera* is also one of the most important topical remedies, an aloe-like plant that has been widely used as a cosmetic moisturizer, toothpaste, food flavoring, and preservative in the pharmaceutical and food fields. It has an antiviral effect and antitumor properties [12,13].

Furthermore, studies have shown that Aloe vera gel successfully treats recurrent aphthous ulcers by reducing pain and speeding healing [12]. The use of Aloe vera led to a reduction in pain intensity and a reduction in the size of aphthous ulcers [14,15]. Comparing Aloe vera with triamcinolone acetonide 0.1% (synthetic corticosteroid), it was found that the latter was superior in reducing pain intensity and the size of aphthous ulcers [16]. Amlexanox 5% has also shown successful results when treating recurrent aphthous ulcers and reducing the intensity of pain and the size of aphthous ulcers [11,17]. When amlexanox 5% was compared with dobetasol (synthetic glucocorticoid corticosteroids), no significant statistical differences were detected between the two products regarding pain intensity and ulcer size [18].

When reviewing the literature, no studies have been found comparing the effectiveness of these two substances in accelerating recurrent aphthous ulcer healing and relieving pain. Therefore, the aim of the present three-arm, randomized, placebo-controlled clinical trial was to compare the effectiveness of Aloe vera gel with that of amlexanox 5% oral paste in treating minor recurrent aphthous ulcers during a five-day observation period. The null hypothesis was that there was no difference between the tested products regarding ulcer size and pain intensity.

## Materials and methods

Study design and settings

This was a three-arm, placebo-controlled, randomized clinical trial. The study was reviewed and approved by the Local Research Ethics Committee at the University of Damascus Dental School (UDDS-212-24092020/SRC-1450) and was funded by the University of Damascus Postgraduate Research Budget (Reference number: 83162306991DEN).

Estimation of the sample size

The sample size was calculated using G* Power 3.1 (Heinrich-Heine-Universität, Düsseldorf, Germany). The sample size was estimated depending on the pain perception score using a visual analog scale (VAS). A minimum total sample size of 60 patients (20 in each group) was found to be sufficient for an alpha of 0.05, power of 80%, and 0.43 as effect size (using the values given in a previous paper [19]).

Patient recruitment and entry in this trial

The sample size was 60 patients with recurrent aphthous ulcers who visited the Department of Oral Medicine at the Faculty of Dentistry at Damascus University. Patients were selected according to the specific inclusion and exclusion criteria. The following criteria were utilized to select the patients with minor recurrent aphthous ulcers: age range 15 to 25 years, presenting with single minor recurrent aphthous ulcers of less than 48 h duration. Exclusion criteria comprised pregnancy and lactation, non-steroidal anti-inflammatory drugs (NSAIDs) consumption, immune modulatory agents, systemic antibiotics, patients on any other intra-oral topical medication with bleeding disorders and corticosteroid therapy, and ulcers as manifestations of systemic diseases. After written informed consent was obtained, the patients were randomly assigned to either Aloe vera, amlexanox, or the control group using a block of random numbers generated by an assistant by Excel 2007 (Microsoft Corporation, Redmond, USA).

The first experimental group: the Aloe vera group

**Figure 1:**
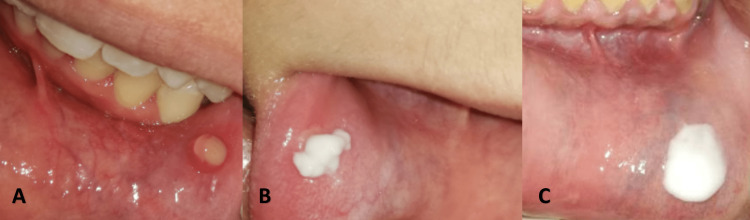
Application of the medication in the three groups. A: Aloe vera gel, B: 5% amlexanox oral paste, and C: the placebo gel.

Twenty patients received the Aloe vera gel (G.U.M® Canker-X®, Sunstar Americas, Schaumburg, USA). On the first day of the trial and after measuring the ulcer size, patients in this group were instructed to apply Aloe vera gel directly to the ulcer three times per day (after meals and before bedtime) using a cotton stick (Figure 1A).

The second experiment group: the amlexanox group

Twenty patients received 5% amlexanox oral paste (Lexanox™ Oral Paste, McLeod's Pharmaceutical Company Limited, Mumbai, India). On the first day of the trial and after measuring ulcer size, patients in this group were instructed to apply 5% Amlexanox oral paste on the ulcer four times a day (after meals and bedtime). The application of the amlexanox oral paste is shown in Figure 1B. 

The control group

Twenty patients received a placebo paste (saline + starch). The placebo was prepared at the Department of Oral Medicine, Faculty of Dentistry at Damascus University by mixing saline with starch in a ratio of three to one and then packing it in sterile containers similar to the sterile containers in which Aloe vera and amlexanox were placed in it for blinding [20]. On the first day of the trial and after recording ulcer size, patients in this group were directed to apply the placebo directly onto the ulcer four times a day (after meals and before bedtime). The application of the placebo paste is given in Figure 1C. Patients were advised that if any allergic reactions occur, they should discontinue the use of the drug and inform the investigator immediately.

Outcome measures: ulcer size reduction and pain perception

Ulcer Size Reduction

**Figure 2:**
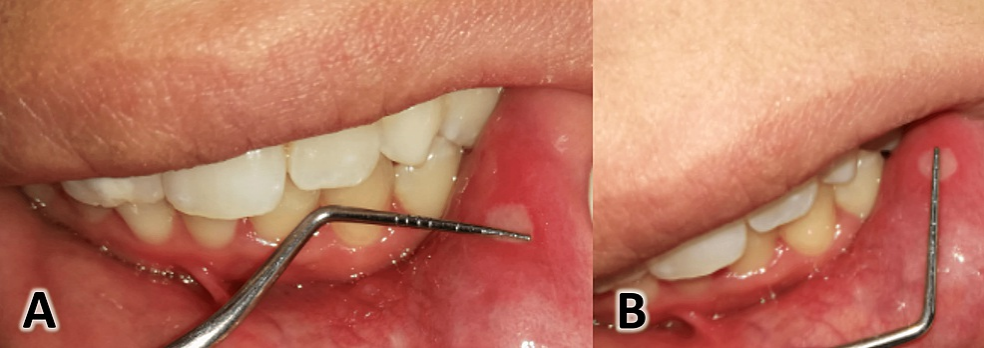
The ulcer size measurement procedure. A: Measuring the maximum diameter of the ulcer. B: measuring the perpendicular diameter to the first one.

Ulcer size was recorded for each treatment period (day 0, day 3, and day 5) using a calibrated William’s periodontal probe with millimeter markings. The procedure was performed by taking two measurements, the first being the maximum diameter of the ulcer and the second being the diameter perpendicular to it, and then multiplying the two measurements to get the ulcer area in square millimeters (Figure 2) [9,21]. Each measurement was taken twice, and the average value was calculated. To evaluate the success rate for each treatment, change in ulcer size (ulcer healing) was assessed on day 3 and day 5 using the same measurement method as on day 0.

Pain Perception

The effect of pain reduction was assessed for each patient using a numeric rating scale (NRS). A line of 10 cm in length was used, and the patient was asked to select the number which best reflected her/his state for the following assessment times: day 0, day 3, and day 5. Patients' responses ranged from (0): no pain, to (10): the highest pain level. This numeric rating scale was converted into a 4-point Likert scale as follows: 0, no pain; (greater than 0 and less than 4), mild pain; greater than or equal to four and less than 7, moderate pain; and greater than or equal to 7, severe pain.

Statistical analysis

SPSS® program Version 13.0 (SPSS Inc., Chicago, USA) was used for statistical analysis. The Kolmogorov-Smirnov test was used to examine the normality of the distributions. One-way analysis of variance (ANOVA) or its alternative nonparametric test (i.e., Kruskal-Wallis test) was utilized to compare the three groups. For post-hoc pairwise comparisons, the Bonferroni test or its alternative nonparametric test (i.e., the Mann-Whitney test) was applied. The level of significance was set at 0.05.

## Results

Basic sample characteristics

**Figure 3:**
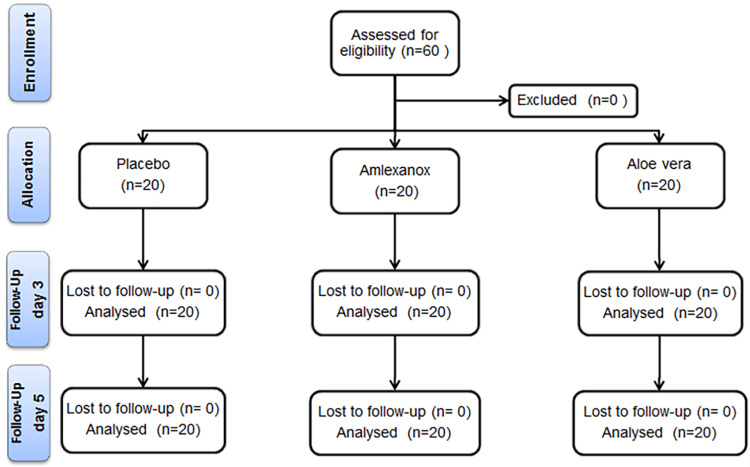
Consolidated Standards of Reporting Trials (CONSORT) flow diagram of patients' recruitment, follow-up, and entry into data analysis.

Sixty patients (33 females, 27 males; mean age ± SD: 20.3 ± 2.4 years) participated in this trial, with 20 patients in each group. Regarding gender distribution, there were 10 males (50.0%) and 10 females (50.0%) in the Aloe vera group, eight males (40.0%) and 12 females (60.0%) in the amlexanox group, and nine males (45.0%) and 11 females (55.0%) in the control group. The age ranges were 16-24 years, 16-24 years, and 15-24 years in the Aloe vera, the amlexanox, and the control groups, respectively. There was no patient withdrawal from the study; consequently, all 60 patients were included in the data analysis (Figure 3).

Ulcer size

The mean ulcer sizes on the first day were 12.70 mm2 ± 3.08, 14.35 mm2 ± 3.90, and 13.10 mm2 ± 2.43 for the Aloe vera, amlexanox, and the control groups, respectively. There were no statistically significant differences in the mean values ​​before treatment between the three groups.

The mean ulcer sizes on the third day were 5.50 mm2 ± 1.54, 8.15 mm2 ± 2.94, and 9.35 mm2 ± 1.98 for the Aloe vera, amlexanox, and the control groups, respectively. On the fifth day, the mean ulcer size was 1.60 mm2 ± 0.36, 4.05 mm2 ± 2.56, and 6.20 mm2 ± 1.67 for the Aloe vera, amlexanox, and the control groups, respectively.

Generally, there were statistically significant differences in the mean values of the aphthous sizes between the three groups on the third and fifth days (Table 1). Pairwise comparisons revealed no statistically significant differences in the mean ulcer size on the third day between the amlexanox and the control groups. The mean ulcer sizes on the third and fifth days in the Aloe vera group were smaller than those in the amlexanox and the control groups. The mean ulcer size, on the fifth day, in the amlexanox group was smaller than that in the control group (Table 2).

**Table 1:** Descriptive statistics of aphthous ulcer size (in mm2) in the three groups at the three assessment times (N=60) N: number of patients; SD: standard deviation; SE: standard error; Min: minimum; Max: maximum; Ϯ employing one-way analysis of variance (ANOVA); * a statistically significant difference at P<0.001; NS: non-significant

Time	Groups	Mean	SD	SE	Min	Max	P-value^Ϯ^	Significance
Day 0	Aloe vera	12.70	3.08	0.69	9	20	0.242	NS
	Amlexanox	14.35	3.90	0.87	9	24		
	Placebo	13.10	2.43	0.54	9	16		
Day 3	Aloe vera	5.50	1.54	0.34	4	9	P<0.001	*
	Amlexanox	8.15	2.94	0.66	4	15		
	Placebo	9.35	1.98	0.44	6	12		
Day 5	Aloe vera	1.85	1.60	0.36	0	4	P<0.001	*
	Amlexanox	4.05	2.56	0.57	0	9		
	Placebo	6.20	1.67	0.37	4	9		

**Table 2:** Descriptive statistics of aphthous ulcer size (in mm2) in the three groups at the three assessment times (N=60) SE: standard error; Ϯ Bonferroni was used to detect any significant difference between the groups; *There was a statistically significant difference at P > 0.05; **There  was a statistically significant difference at P<0.001; NS: non-significant difference

Time	Group (I)	Group (J)	Mean Diff (I-J)	SE	P-valueϮ	Significance
Day 3	Aloe Vera	Amlexanox	-2.65	0.71	0.001	*
		Placebo	-3.85	0.71	P<0.001	**
	Amlexanox	Placebo	-1.20	0.71	0.284	NS
Day 5	Aloe Vera	Amlexanox	-2.20	0.63	0.003	*
		Placebo	-4.35	0.63	P<0.001	**
	Amlexanox	Placebo	-2.15	0.63	0.004	*

Pain perception

The mean changes in pain perception on the third day were -3.90 mm ± 1.12, -2.90 mm ± 0.72, and -2.10 ± 0.55 for the Aloe vera, the amlexanox, and the control groups, respectively. The mean changes in pain perception on the fifth day were -6.05 mm ± 1.43, -5.05 mm ± 1.05, and -4.05 mm ± 0.94 for the Aloe vera, the amlexanox, and the control groups, respectively. There were statistically significant differences in the mean changes in pain perception on the third and fifth days between the three groups, in general (Table 3). Pairwise comparisons revealed that the mean reduction in pain perception on the third and fifth days was greater in the Aloe vera group compared to the amlexanox and control groups, whereas the mean reduction of pain levels on the third and fifth days was greater in the amlexanox group compared to the control group (Table 4).

**Table 3:** Descriptive statistics of changes observed in pain perception in each group (N=60) N: number of patients; SD: standard deviation; SE: standard error; Min: minimum; Max: maximum; Ϯ employing one-way analysus of variance (ANOVA); *significant difference

Time	Group	Mean	SD	SE	Min	Max	P-valueϮ	Significance
Day 3	Aloe vera	-3.90	1.12	0.25	-6	-2	P<0.001	*
	Amlexanox	-2.90	0.72	0.16	-4	-2		
	Placebo	-2.10	0.55	0.12	-4	-1		
Day 5	Aloe vera	-6.05	1.43	0.32	-8	-3	P<0.001	*
	Amlexanox	-5.05	1.05	0.23	-6	-3		
	Placebo	-4.05	0.94	0.21	-7	-3		

**Table 4:** Post-hoc tests for pairwise comparisons of the observed changes in pain perception at the third-day and fifth-day assessment times SE: standard error; Ϯ Bonferroni was used to detect any significant difference between the groups; *There was a statistically significant difference at P > 0.05; **There was a statistically significant difference at P<0.001

Time	Group (I)	Group (J)	Mean Diff (I-J)	SE	P-value^Ϯ^	Significance
Day 3	Aloe vera	Amlexanox	-1.00	0.26	0.001	*
		Placebo	-1.80	0.26	P<0.001	**
	Amlexanox	Placebo	-0.80	0.26	0.011	*
Day 5	Aloe vera	Amlexanox	-1.00	0.37	0.026	*
		Placebo	-2.00	0.37	P<0.001	**
	Amlexanox	Placebo	-1.00	0.37	0.026	*

## Discussion

The main goals of treating recurrent aphthous ulcers are to relieve pain, reduce ulcer size and duration, and restore normal oral function. Secondary goals include reducing the frequency and severity of the recurrence of these ulcers [22]. A wide range of treatments has been tried, from topical agents to systemic medications, physical methods, and natural and home remedies [23].

The current study aimed to evaluate the effectiveness of both Aloe vera gel and amlexanox 5% oral paste in reducing ulcer size and pain. Ulcer size in group one and group two showed significant improvement between day 0 and day 3, day 0 and day 5, and day 3 and day 5. There was a significant difference between Aloe vera gel and amlexanox 5% paste in reducing ulcer size on the fifth day, as the ulcer size reduction was greater in the Aloe vera group. The wound-healing effect of Aloe vera can be attributed to its ability to increase the migration of epithelial cells. Glucomannan (a polysaccharide-rich in mannose) and gibberellin (a growth hormone) in Aloe vera extract promote collagen synthesis, and gibberellin interacts with growth factor receptors on fibroblasts, stimulating their activity and proliferation. Aloe vera also increases the synthesis of hyaluronic acid and dermatan sulfate in the granulation tissue of the wound, which promotes wound healing. Aloe vera also contains a series of components, such as acemannan, that have wound-healing potential by promoting the repair process and proliferation of epithelial cells by stimulating factors contributing to wound repair, including fibroblasts and collagen [12,15]. The antioxidant components in Aloe vera also enhance the anti-inflammatory effects by inhibiting the production of reactive oxygen metabolites, thus preventing oxidative stress [24]. Aloe vera gel also affects the healing process and normal microbiota of recurrent aphthous ulcers. It can reduce the abundance of harmful oral bacteria, including *Actinomyces*, *Granulicatella*, and *Peptostreptococcus*. Therefore, it can improve the quality of life for patients with recurrent aphthous ulcers [15].

Also, the values ​​of aphthous ulcer size on the fifth day in the group treated with amlexanox were smaller than in the group treated with a placebo. Amlexanox is known to potentiate its effect by increasing the intracellular concentration of adenosine monophosphate (AMP) or by inhibiting the influx of calcium into cells [25]. Both the first group and the second group showed a significant decrease in NRS scores between day 0 and day 3, day 0 and day 5, and day 3 and day 5, but when comparing the two groups, Aloe vera gel showed a significant difference in reducing pain on the fifth day, and this indicates that the Aloe vera has a significant therapeutic effect in reducing the intensity of pain, and this is consistent with the study of Babaee et al., where it was found that Aloe vera gel is effective in relieving ulcer pain [26]. According to the literature, Aloe vera gel inhibits the cyclooxygenase pathway and reduces the production of prostaglandin E2 from arachidonic acid. Recently, a new anti-inflammatory compound called C-glucosyl chromone was isolated from gel extracts that help relieve pain [27]. Also, the values ​​of pain volume on the fifth day in the group treated with amlexanox were smaller than in the group treated with a placebo. Amlexanox is a topical anti-inflammatory and anti-allergic drug that reduces the release of histamine and leukotriene from mast cells, neutrophils, and mononuclear cells. Histamines and leukotrienes are vasoactive inflammatory molecules known to act by increasing vascular permeability and causing inflammation in affected tissues [28].

Limitations

One limitation of the current study is that the application of treatment depended on the patient compliance with the instructions. Therefore, patient adherence to the given instructions was not assessed. The observation period was confined to five days only. This period could have been extended more, and insight into the level of recurrence could have been established. Other patient-reported outcomes, such as mastication difficulties or speech problems, should be evaluated in future research. The oral health-related quality of life could be another area of investigation in this context.

## Conclusions

Significant differences were observed between the Aloe vera, the amlexanox, and the placebo groups regarding the mean ulcer size after five days of observation. The Aloe vera gel and amlexanox effectively accelerated the healing of aphthous ulcers, with the Aloe vera gel group showing a greater decrease in ulcer size. Significant differences were also observed between the Aloe vera, the amlexanox, and the placebo groups regarding pain intensity at five days following gel application. Aleo vera and amlexanox effectively reduced the perceived pain, with the Aleo vera group showing a greater reduction in pain compared to the amlexanos group.
